# Synergistic Effect of Graphene/Silver Nanowire Hybrid Fillers on Highly Stretchable Strain Sensors Based on Spandex Composites

**DOI:** 10.3390/nano10102063

**Published:** 2020-10-19

**Authors:** Tan Thong Vo, Hyeon-Jong Lee, Sang-Yun Kim, Ji Won Suk

**Affiliations:** 1 School of Mechanical Engineering, Sungkyunkwan University, Suwon, Gyeonggi-do 16419, Korea; votanthong1994@gmail.com (T.T.V.); whdwhd1110@naver.com (H.-J.L.); kimsangyun94@gmail.com (S.-Y.K.); 2 SKKU Advanced Institute of Nanotechnology (SAINT), Sungkyunkwan University, Suwon, Gyeonggi-do 16419, Korea

**Keywords:** stretchable strain sensors, composites, graphene, silver nanowires, human motion detection

## Abstract

Embedding conductive nanomaterials into elastomeric polymer matrices is one of the most promising approaches for fabricating stretchable strain sensors capable of monitoring large mechanical movements or deformation through the detection of resistance changes. Here, hybrid fillers comprising graphene and silver nanowires (AgNWs) are incorporated into extremely stretchable spandex to fabricate strain sensors. Composites containing only graphene and those containing the graphene/AgNW hybrid fillers are systematically investigated by evaluating their electrical and mechanical properties. The synergistic effect between graphene and AgNWs enable the strain sensors based on the composites to experience a large strain range of up to 120%, and low hysteresis with a high gauge factor of 150.3 at a strain of 120%. These reliable strain sensors are utilized for monitoring human motions such as heartbeats and body movements. The findings of this study indicate the significant applicability of graphene/AgNW/spandex composites in future applications that demand high-performance stretchable strain sensors.

## 1. Introduction

There has been an increasing interest in the development of high-performance strain sensors for a wide variety of future applications such as human motion and health monitoring [1,2,3], wearable electronics [4,5,6], soft robotics [7,8,9], and structural health monitoring [10,11]. The strain sensors suitable for these applications need to be soft and stretchable to detect large mechanical movements or deformation. Conventional strain sensors based on thin metal foils have poor stretchability, which limits their potential for the abovementioned future applications. Previous studies have reported the structuring of conductive and stiff films with wavy or wrinkled geometries for the purpose of improving stretchability [12,13,14]. However, this approach requires complicated fabrication processes such as sequential patterning and etching.

Recently, electrically conductive polymer composites have been intensively investigated as a solution to realize stretchable strain sensors, because they possess superior physical properties and high scalability in manufacturing with low costs [15]. Stretchable and conductive polymer composites are typically fabricated by incorporating conductive nanomaterials as fillers into elastomeric polymers such as poly(dimethylsiloxane) (PDMS) [16], polyurethane (PU) [17], poly(styrene-butadiene-styrene) (SBS) [18], and natural rubber (NR) [19]. Metal- or carbon-based nanomaterials with various dimensionalities have been investigated as conductive fillers for composites; examples of these nanomaterials include zero-dimensional (0D) nanoparticles (NPs) [20] and carbon black (CB) [21]; one-dimensional (1D) nanowires (NWs) [22] and carbon nanotubes (CNTs) [23]; and two-dimensional (2D) nanoplates [24] and graphene [25]. Electrical connections of the conductive nanofillers in polymer matrices are achieved by increasing the filler concentration beyond the percolation threshold. However, increasing the filler concentration can also increase the stiffness and brittleness of the composites [26], which are undesirable traits for stretchable strain sensors. To maintain the mechanical properties of stretchable polymers, the amount of conductive fillers should be controlled, while maintaining an appropriate electrical conductivity. Thus, a conductive polymer composite with a low percolation threshold is suitable for stretchable strain sensors.

Combining two different types of fillers has been a promising method to obtain lower percolation thresholds in composites with an enhanced performance. Nanomaterials featuring 1D or 2D geometries have been extensively used as one or both of the bifillers, owing to their unique geometries with high aspect ratios [27,28]. Hybrid fillers containing 1D and 2D materials, such as CNT/graphene [29,30] and silver nanowire (AgNW)/graphene oxide (GO) [27], generate a higher electrical conductivity while using a low amount of fillers, for instance. This is because one type of filler bridges the gaps within the other type of filler due to the high-aspect-ratio morphology, thereby establishing robust conductive pathways with lesser loading of fillers. Additionally, the use of hybrid fillers yields a better dispersion of fillers in polymer matrices. It has been shown that hybrid fillers containing CNTs and exfoliated graphene prevent the agglomeration of CNTs and the restacking of graphene flakes in the polymer matrix [30]. Similarly, GO, which is an insulating graphene-based material, acts as a dispersion agent to prevent the agglomeration of AgNWs [27]. Although graphene and AgNWs have been evaluated for synthesizing polymer composites such as epoxy [28], very few studies have focused on strain sensors employing stretchable polymer composites fabricated by mixing conductive graphene and AgNWs with highly stretchable polymers such as spandex.

Spandex is a urethane-containing polymer with alternating soft and hard segments. Owing to its unique chemical structures, spandex features an extremely high stretchability of over 700% and superior elastic recovery [31]; hence, it is a promising material for stretchable strain sensors based on polymer composites. Here, we incorporate hybrid fillers containing graphene flakes and AgNWs into spandex, to fabricate highly stretchable strain sensors. The synergy between graphene and AgNWs provides higher electrical conductivity, higher stretchability, and lower hysteresis, as compared to the composite containing graphene alone. The strain sensors based on the composite containing hybrid fillers yields reliable resistance responses up to a strain of 120%, with high sensitivity. Additionally, we establish a simple method to eliminate the undesirable shoulder peaks that typically appear in the resistance-strain responses of stretchable polymer composites. These high-performance stretchable strain sensors are utilized for the detection of human motions such as heartbeats and the movements of a wrist, finger, and knee joint.

## 2. Materials and Methods

### 2.1. Fabrication of Spandex Composites

Reduced graphene oxide (rGO, TGF600, Grapheneall, Suwon, Korea) and AgNWs (Flexiowire 2020, Flexio Co., Ltd., Daejeon, Korea) were used as the conductive fillers in the composites. Spandex fibers (Creora, Hyosung, Seoul, Korea) were soaked in petroleum ether for 30 min to remove the cover layers. Thereafter, the fibers were placed in ethanol for 30 min to eliminate any residual petroleum ether. After cleaning, the fibers were dried in a convection oven. The clean spandex fibers (500 mg) were dissolved in *N*,*N*-dimethylacetamide (DMAc, 10 mL) at 80 °C by stirring for 2 h.

A graphene suspension (1.2 mg/mL) was prepared by sonicating the graphene powders in *N*,*N*-dimethylformamide (DMF) for 8 h. A small amount of the spandex solution was added into the graphene suspension to obtain a better dispersion of graphene in the composite. This pre-mixture of graphene and spandex was stirred at 400 rpm at 80 °C for 12 h.

Graphene/AgNW (G/AgNW) hybrid fillers were prepared by adding an AgNW solution (0.5 wt% in isopropyl alcohol) to the graphene suspension. Furthermore, a small amount of the spandex solution was added into the G/AgNW suspension. The amount of AgNWs was adjusted to obtain different ratios of AgNWs to graphene. This pre-mixture of graphene, AgNWs, and spandex was stirred at 400 rpm at 80 °C for 12 h.

The pre-mixture solution of graphene or G/AgNW fillers with spandex was mixed with the spandex solution by stirring at 400 rpm at 80 °C for 4 h. Finally, the solution was poured into a mold and dried at 60 °C to form composite films with a thickness of approximately 50 μm to be used in the strain sensors.

### 2.2. Characterization of the Materials

The surface morphologies of graphene, AgNWs, and the composite films were observed using scanning electron microscopy (SEM, JSM-7600, Jeol, Tokyo, Japan). Raman spectroscopy (ALPHA300M with a 532-nm wavelength laser, WiTec, Ulm, Germany) and X-ray photoelectron spectroscopy (XPS, ESCALAB-250 with monochromated Al K_α_ radiation, Thermo-Scientific, Waltham, MA, USA) were used to characterize the graphene powders. During the XPS analysis, the C 1s core-level spectra were deconvoluted via Gaussian–Lorentzian functions after the background signal was subtracted using the Shirley background model. The asymmetric Doniach–Sunjic line shape was used for the sp^2^-hybridized carbon [32,33,34,35], and the Gaussian–Lorentzian product formula was applied for the other components in the deconvolution process [36,37].

### 2.3. Fabrication and Testing of Strain Sensors

The composite films were cut into rectangular strips with a width of 1 cm to fabricate the strain sensors. Copper tapes were attached at the two ends of the composite film strips to act as electrodes. Two copper tapes were attached on both upper and lower sides of each end of the composite strip. Silver paste was positioned between the composite strip and the copper tape to ensure robust electrical contacts. Mechanical and electro-mechanical testing were performed using a universal testing machine (UTM). The copper tape parts of the composite strip were gripped by the UTM. The applied force was measured using a precise load cell (LRM-50N, ALGOL Instrument Co., Ltd., Taoyuan City, Taiwan). The thickness of the composite film was measured using a micrometer (293–821, Mitutoyo Corporation, Kawasaki-shi, Japan) with a resolution of 1 μm to estimate mechanical stress. The changes in the resistance of the strain sensors, which were caused by stretching and releasing them using the UTM, were monitored. Electrical measurements were performed and recorded using an external circuit with a multifunction input/output device, by employing the LabVIEW software (National Instrument, Austin, TX, USA). The resolution of the resistance measurement was 0.01 Ω. The relative change in resistance was calculated using the following equation:

(1) $$\frac{\Delta R}{R_{0}}=\frac{R-R_{0}}{R_{0}}$$

where *R*_0_ is the initial resistance of the strain sensor, and *R* is the resistance of the sensor under an applied strain. The gauge factor (GF) of the strain sensors was defined as follows:

(2) $$\mathrm{GF}=\frac{\Delta R/R_{0}}{\varepsilon }=\frac{\Delta R/R_{0}}{\Delta L/L_{0}}$$

where *ε* is the applied strain, *L*_0_ is the gauge (initial) length between two electrodes, and Δ*L* is the change in length. Mechanical hysteresis (H_M_) of the composites for a complete loading–unloading cycle was quantified based on the stress–strain curve, using the following equation [38]:

(3) $$H_{M}\left(\%\right)=\frac{\left|A_{S}-A_{R}\right|}{A_{S}}\times 100$$

where *A*_S_ and *A*_R_ are the areas under the stretching and releasing curves of the stress–strain curves, respectively.

## 3. Results and Discussion

### 3.1. Graphene and AgNWs as Hybrid Fillers

**Figure 1:**
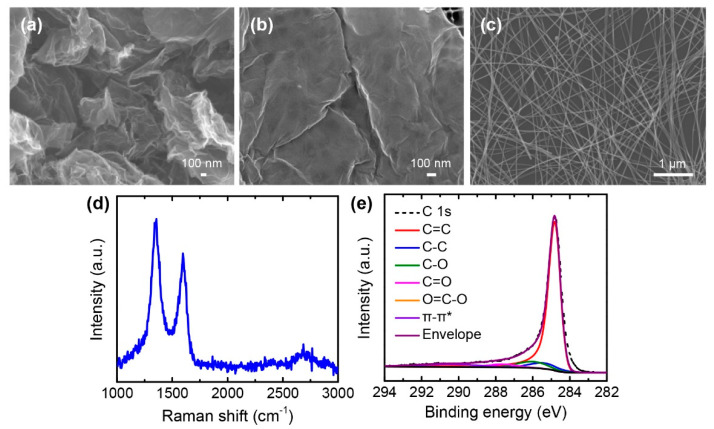
Characterization of reduced graphene oxide (rGO) and silver nanowires (AgNWs). (**a**,**b**) Scanning electron microscopy (SEM) images of: (**a**) as-received rGO powders; (**b**) Sonicated rGO powders; (**c**) SEM image of AgNWs placed on a silicon substrate; (**d**) Raman spectrum of as-received rGO powders; (**e**) X-ray photoelectron spectroscopy (XPS) C 1s core-level spectra of as-received rGO powders.

As-received graphene powders were crumpled and agglomerated with many wrinkles, ripples, and voids, as shown in Figure 1a. This hindered an appropriate dispersion of graphene in the solvents and polymer matrix. Therefore, rather than using additional materials, the surface morphology of the graphene powders was modified to improve dispersion. A simple sonication treatment was used as an effective method to transform crumpled shapes into flat surfaces [39]. The strong shear stresses and cavitation during sonication treatment altered the crumpled morphology of the graphene powders. DMF was chosen as the solvent to disperse graphene flakes because it possesses the appropriate Hansen solubility parameters and surface energy [40]. Moreover, DMF has a relatively low boiling point, as compared to other solvents for graphene; this facilitates the formation of composite films during the drying process. After treating the crumpled graphene powders in a bath sonicator for 8 h, flattened graphene flakes were obtained, as presented in Figure 1b. Thus, a better dispersion of graphene in DMF and the spandex solution was obtained.

AgNWs were also used as conductive fillers due to their excellent electrical conductivity and high-aspect-ratio geometries. Figure 1c presents the AgNWs dispersed on a silicon substrate. These AgNWs have an average diameter of about 25 nm and an average length of about 20 μm, which indicates their extremely high aspect ratio of about 800.

Raman spectroscopy and XPS were employed to characterize the quality of graphene powders after the sonication treatment. The Raman spectrum indicated the G band at around 1350 cm^−1^ and the D band at around 1595 cm^−1^, with a slight increase in the 2D band at around 2680 cm^−1^, thus demonstrating the typical fingerprint of rGO (Figure 1d) [41]. Detailed chemical structures of the graphene flakes were analyzed by deconvoluting the C 1s spectrum obtained from XPS (Figure 1e). The deconvoluted peaks corresponded to the sp^2^-hybridized carbon (C=C) at 284.7 eV, sp^3^-hybridized carbon (C-C) at 285.5 eV, C-O at 286.1 eV, C=O at 287.6 eV, O=C-O at 288.6 eV, and π-π* transition at 290.6 eV [36,37,42]. The reduced oxygen functional groups, compared to GO, resulted in an estimated C/O ratio of 6.3, which was similar to that of typical rGO [41].

### 3.2. Spandex Composite Films

**Figure 2:**
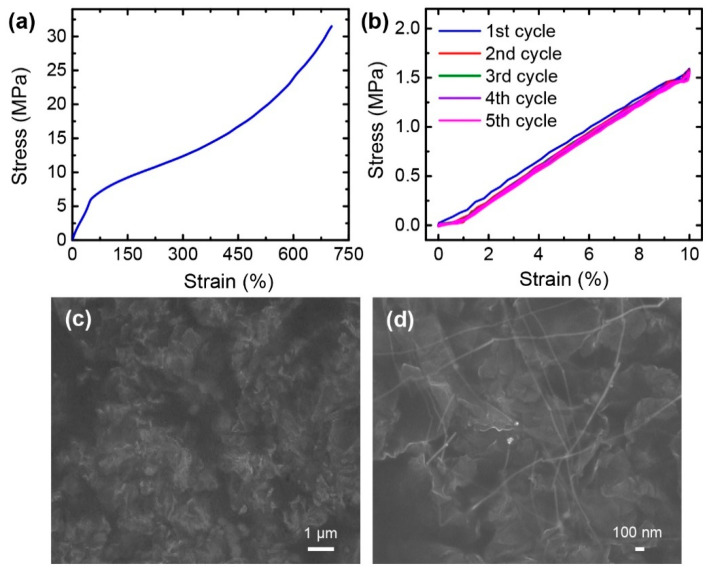
(**a**) Stress–strain curve of the neat spandex film; (**b**) Stress–strain curves of the neat spandex film under stretching and releasing cycles with a strain of 10%; (**c**,**d**) SEM images of the surfaces of the: (**c**) Graphene/spandex (G/SpX); (**d**) Graphene/AgNW/spandex (G/AgNW/SpX) composite films.

Spandex was selected as a polymer matrix owing to its extremely high stretchability and superior elastic recovery [31]. A neat spandex film without any conductive fillers exhibited a fracture strain of 703% (Figure 2a). Furthermore, the spandex film exhibited good mechanical stability with low hysteresis under the cyclic stretching and releasing tests (Figure 2b). These results indicate that spandex is a promising candidate for the polymer material used in highly stretchable strain sensors.

After modifying the morphology of the graphene powders, they were well-dispersed in the spandex matrix, as shown in the SEM image of the composite surface in Figure 2c. However, the graphene flakes were highly wrinkled and crumpled in the polymer matrix. This can be attributed to the strong interaction forces of graphene due to its large surface area and extreme flexibility [43,44,45]. Figure 2d presents the surface of the graphene/AgNW/spandex (G/AgNW/SpX) composite film. The graphene flakes and AgNWs were well-dispersed in the spandex matrix. Additionally, the AgNWs maintained their high-aspect-ratio geometries in the polymer, thus connecting several graphene flakes. Compared to the graphene/spandex (G/SpX) composite, the graphene flakes in the G/AgNW/SpX composite film exhibited a smoother morphology and less crumpled shapes. This is because the AgNWs acted as spacers between the graphene flakes, reducing the interaction between graphene layers [30]. Simultaneously, the graphene flakes prevented the agglomeration of AgNWs [27]. This enhanced the dispersion of graphene flakes and AgNWs in the composite, which is beneficial for improving the electrical and mechanical properties of the composite.

**Figure 3:**
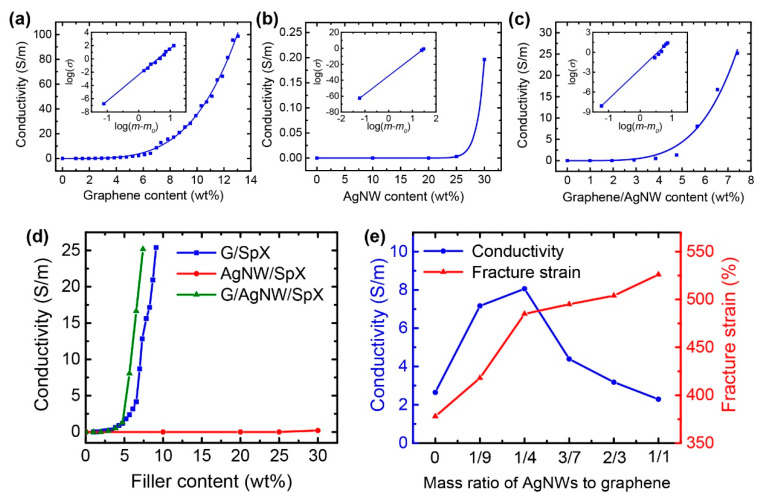
(**a**–**d**) Electrical conductivity of composite films as a function of filler loading; The insets in (**a**–**c**) show the log(*σ*) versus log(*m*−*m*_0_); The G/AgNW/SpX composite in (**c**) had a mass ratio of AgNWs to graphene of 1/4; (**d**) Comparision of the electrical conductivity of composite films; (**e**) Electrical conductivity and fracture strain of the G/AgNW/SpX composite as a function of the mass ratio of AgNWs to graphene, with a total filler concentration of 5.66 wt%.

To determine the percolation threshold, a series of composites with various graphene and AgNW contents were fabricated, and their electrical conductivities were measured, as shown in Figure 3a–d. The electrical conductivity of composites (*σ*) obeys a power-law relationship based on the classical percolation theory [46]:

(4) $$\sigma =\sigma_{0}{\left(m-m_{0}\right)}^{t}$$

where *σ*_0_ is the electrical conductivity of a conductive filler, t is the critical exponent, *m* is the filler content, and *m*_0_ is the percolation threshold. Via fitting the equation with the experimental data, the G/SpX composite exhibited a percolation threshold of 1.48 wt% (Figure 3a). On the contrary, the AgNW/SpX composite did not exhibit conductivity even at 20 wt% (Figure 3b); this indicates that it requires a significant amount of AgNWs to realize conductive spandex composites. Although AgNWs have high electrical conductivities, composites containing AgNWs exhibited reduced stretchability with increasing amounts of AgNWs [47]. Therefore, the addition of 2D graphene to 1D AgNWs can yield better electrical and mechanical performance of the composites, with a minimal use of AgNWs and at lower costs. Figure 3c,d show that the G/AgNW hybrid filler provided higher electrical conductivity of the composite at a given filler loading amount, compared to the G/SpX and AgNW/SpX cases. This synergistic effect of 2D graphene and 1D AgNWs occurred because one filler bridged the other, which formed the robust conductive paths in the composites, as shown in Figure 2d.

The synergistic effect of graphene and AgNWs on the electrical and mechanical properties of the composite film was further investigated by changing the mass ratio of AgNWs to graphene. Figure 3e depicts the electrical conductivity and fracture strain of the G/AgNW/SpX composite films for different mass ratios of AgNWs to graphene ranging from 0 to 1. The total amount of fillers was maintained at 5.66 wt%. There was a sharp increase in the fracture strain and electrical conductivity when the ratio increased up to 1/4. Over this ratio of 1/4, the fracture strain increased slowly, whereas the electrical conductivity decreased as the ratio increased up to 1. Owing to the large specific surface area and a high Young’s modulus of graphene [48,49], its addition can dramatically reduce the stretchability of the composite [50]. Based on this observation, the strain sensors were fabricated at the mass ratio of AgNWs to graphene of 1/4. The G/AgNW/SpX composite with a ratio of 1/4 yielded higher electrical conductivity than the G/SpX composite (Figure 3d); the electrical conductivity of the G/AgNW/SpX composite was 8.06 S/m at 5.66 wt%, whereas that of the G/SpX composite was 2.37 S/m, at the same filler loading.

**Figure 4:**
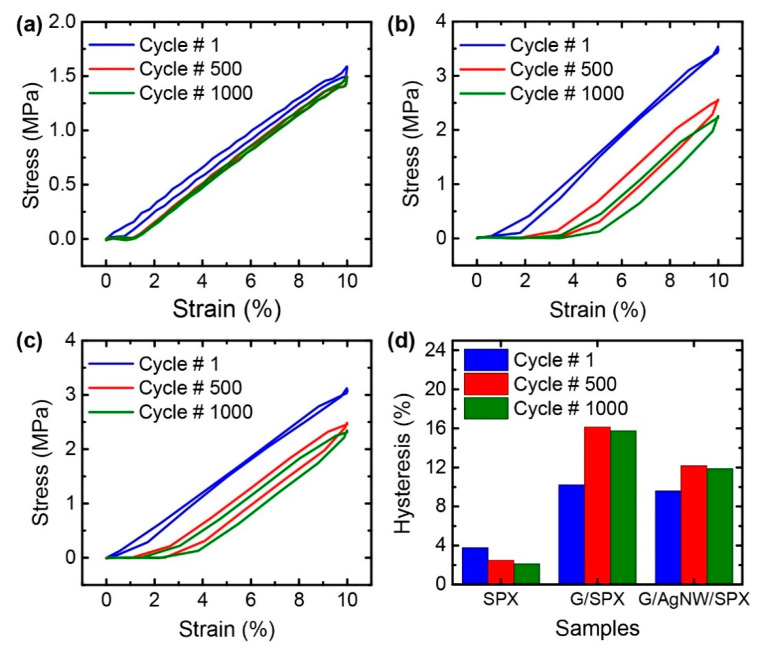
(**a**–**c**) Stress–strain curves of: (**a**) neat spandex; the (**b**) G/SpX; (**c**) G/AgNW/SpX films during the 1st, 500th, and 1000th cycles; (**d**) Mechanical hysteresis (H_M_) obtained from the stress–strain curves for each film.

The H_M_ values of the composite comprising graphene alone and those comprising the G/AgNW hybrids were compared. Figure 4 presents the stress–strain curves for the neat spandex, G/SpX, and G/AgNW/SpX films. The H_M_ values of the neat spandex at the 1st, 500th, and 1000th cycles were 3.77, 2.48, and 2.12%, respectively. When spandex was mixed with graphene flakes, the H_M_ values increased to 10.22, 16.13, and 15.76% for the 1st, 500th, and 1000th cycles, respectively. However, the H_M_ values of the G/AgNW/SpX composite were 9.58, 12.16, and 11.87% for the 1st, 500th, and 1000th cycles, respectively; these values were lower than those of the G/SpX composite. This indicates that the hybrid fillers contributed toward the fabrication of composite films featuring lower hysteresis.

### 3.3. Resistance-Strain Responses of the Stretchable Strain Sensors

**Figure 5:**
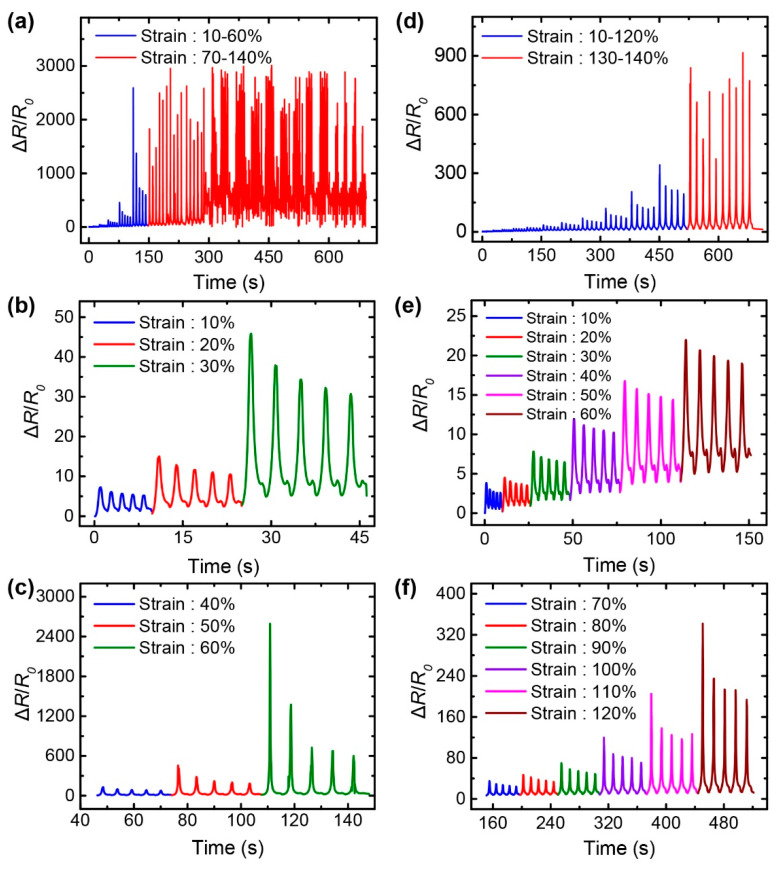
Resistance responses of the: (**a**–**c**) G/SpX; (**d**–**f**) G/AgNW/SpX composite strain sensors under various strain levels; (**a**,**d**) Relative change in resistance of the sensors at strains ranging from 10- to 140%; (**b**,**c**,**e**,**f**) Detailed resistance-strain responses of the sensors at each strain level.

To evaluate the maximum strain range of the strain sensors, the sensors were repeatedly stretched and released, while increasing the strain from 10 to 140%. Strain sensors employing the G/SpX and G/AgNW/SpX composites were tested at a strain rate of 16.7%/s. Due to the increased separation of fillers, the G/SpX strain sensor exhibited a proportional increase in its relative resistance change as the strain increased up to 60% (Figure 5a–c), thus demonstrating a positive piezoresistive effect. This indicates that the sensor is capable of detecting and quantifying an applied strain. However, the sensor was unstable when the strain exceeded 70%, which implies that the percolation network composed of graphene flakes in the spandex matrix was broken and could not be reformed at high strain levels. Conversely, the G/AgNW/SpX strain sensor exhibited reliable resistance responses as the strain increased to 120%; however, these responses were unstable when the strain exceeded 130% (Figure 5d–f). This implies that the combination of graphene and AgNWs provides more stable electrical connections in the polymer matrix, which can be retained even at high strain levels; this is because the long AgNWs bridge the broken graphene flake network at high strains, thereby maintaining the electrical network within the composite.

**Figure 6:**
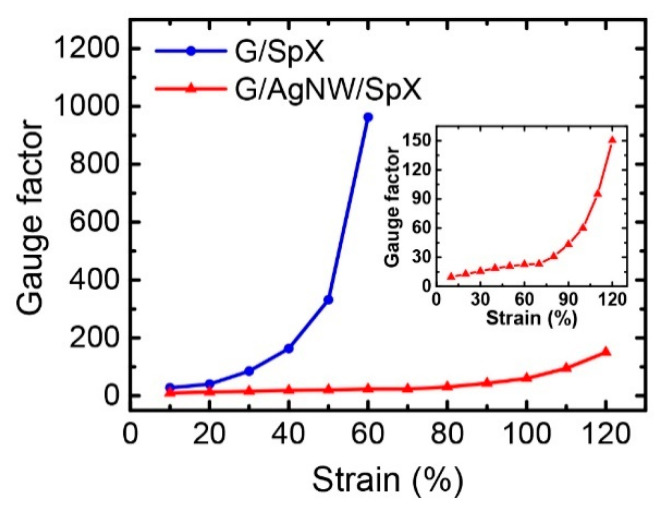
GFs of the G/SpX and G/AgNW/SpX composite strain sensors. The inset shows the detailed GF of the G/AgNW/SpX sensor.

The sensitivity of the sensors was estimated by calculating the GF at each strain level. Figure 6 shows the GF change of the G/SpX and G/AgNW/SpX composite strain sensors. Accompanying increasing strains, the GF increases because the distance between neighboring fillers gradually increases. Occurring at higher strains, there is severe destruction of the conductive network, which results in higher GFs [51,52]. The GF of the G/SpX sensor increased from 27.7 to 963 as the strain increased from 10 to 60%, whereas the GF of the G/AgNW/spandex sensor increased from 9.7 to 150.3 under strain levels ranging from 10 to 120% (Figure 6). Thus, the G/SpX sensor achieved a higher sensitivity than the G/AgNW/SpX sensor. However, the G/AgNW/SpX sensor featured a larger operating strain range, with reasonably high sensitivity.

There have been extensive researches on stretchable strain sensors based on conductive composites. Recently, Want et al., developed a sandwich-based strain sensor fabricated by vacuum filtration of AgNWs/graphene/AgNWs and encapsulation with PDMS [53]. It exhibited a high GF of 1403.7 at a strain of 35%. However, the working strain range was limited to only 35%. Lin et al., used butadiene styrene rubber (SBR)/ NR assembled with functionalized graphene for stretchable strain sensors [54]. They demonstrated a GF of 82.5 and high stretchability of up to 120%. Zheng et al., incorporated CNT/CB hybrid fillers in PDMS for conductive composites [51], showing high stretchability of up to 300% with a limited GF of 13.1. Wu et al., used vertical graphene nanosheets embedded in PDMS, which exhibited a GF of 32.6 with high stretchability of up to 120% [55]. Thus, compared with other works, the G/AgNW/SpX sensor shown in this work demonstrates both high stretchability and high GF.

**Figure 7:**
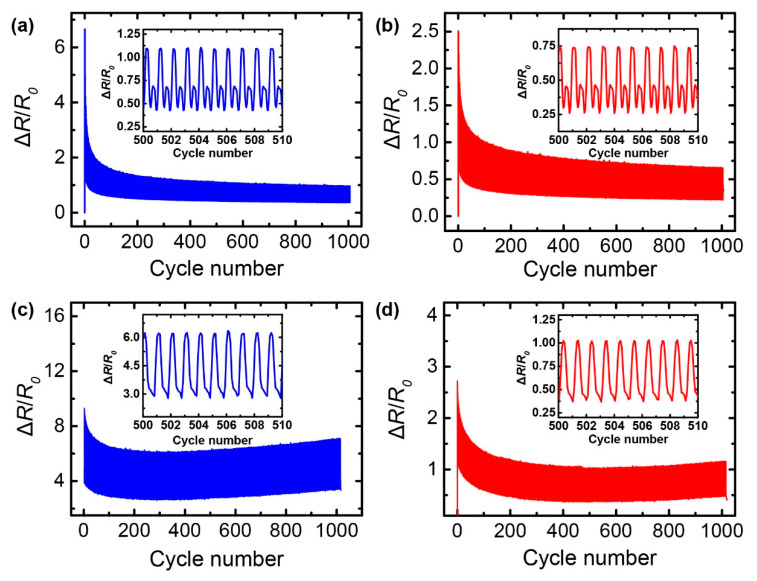
Resistance-strain responses of the: (**a**,**c**) G/SpX; (**b**,**d**) G/AgNW/SpX composite strain sensors under 1000 stretching–releasing cycles with a strain of 10%; (**a**,**b**) Without pre-strain; (**c**,**d**) With 10% pre-strain. The insets show the detailed responses of the sensors.

The resistance-strain responses were characterized for both strain sensors during 1000 cycles, at a strain of 10% and a strain rate of 16.7%/s (Figure 7a,b). Both strain sensors demonstrated similar trends during this cyclic test; in the early stage, there was a sharp increase in the relative resistance change due to the destruction of the electrical network in the polymer matrix, which was caused by the applied strain. Subsequently, the resistance-strain responses gradually stabilized over the cycles, due to the reformation of conductive networks in the composite. However, as shown in the insets of Figure 7a,b, both strain sensors exhibited small shoulder peaks next to the main peaks, showing a negative piezoresistive effect during the releasing process. Although this behavior has not been completely understood, this might be attributed to the bi-directional motion of the filler network affected by the polymer chain mobility and the Poisson’s ratio [38]. When the composite returns to the original state after stretching, the longitudinal distance (along its length) between two neighboring fillers decreases; however, the transverse distance (along its width) between them increases due to Poisson’s effect, while the filler network is irreversibly changed due to the slippage of fillers during the stretching process. To eliminate these undesirable non-monotonic behaviors, a pre-strain was applied to the strain sensor to enable the reformation of the conductive network through tight binding between the fillers and the polymer matrix. Figure 7c,d show the results of the cyclic tests on the strain sensor with a pre-strain of 10%. The shoulder peaks of the resistance-strain responses were eliminated for both strain sensors, as shown in the insets of Figure 7c,d. Furthermore, the resistance-strain response of the G/AgNW/SpX composite subjected to a pre-strain was more leveled off than that of the G/SpX composite. Although the pre-straining of the sensor was not optimized in this study, these findings confirm that this method is capable of achieving stable sensor responses.

**Figure 8:**
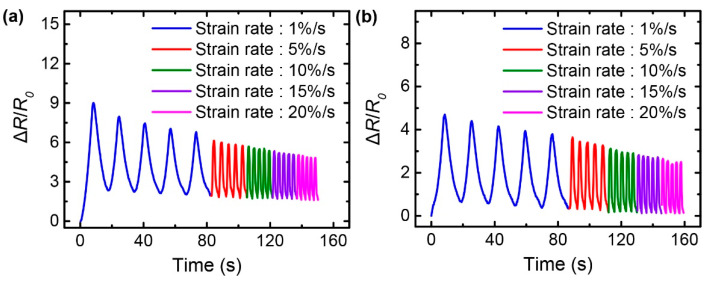
Resistance-strain responses of the: (**a**) G/SpX; (**b**) G/AgNW/SpX composite strain sensors at various strain rates, with a strain of 10%. The pre-strain of 10% was applied to the sensors.

Since stretchable strain sensors can undergo deformations at different rates, the sensor responses at different strain rates are an important aspect that should be considered during applications. The effect of strain rate on the resistance-strain responses was investigated by varying the strain rate from 1 to 20%/s, for the G/SpX and G/AgNW/SpX strain sensors subjected to an applied strain of 10% with a pre-strain of 10% (Figure 8). Both the strain sensors yielded stable responses at all the applied strain rates, thereby exhibiting potential for the detection of diverse mechanical movements or deformation at various speeds.

### 3.4. Human Motion Detection Using the Strain Sensors

**Figure 9:**
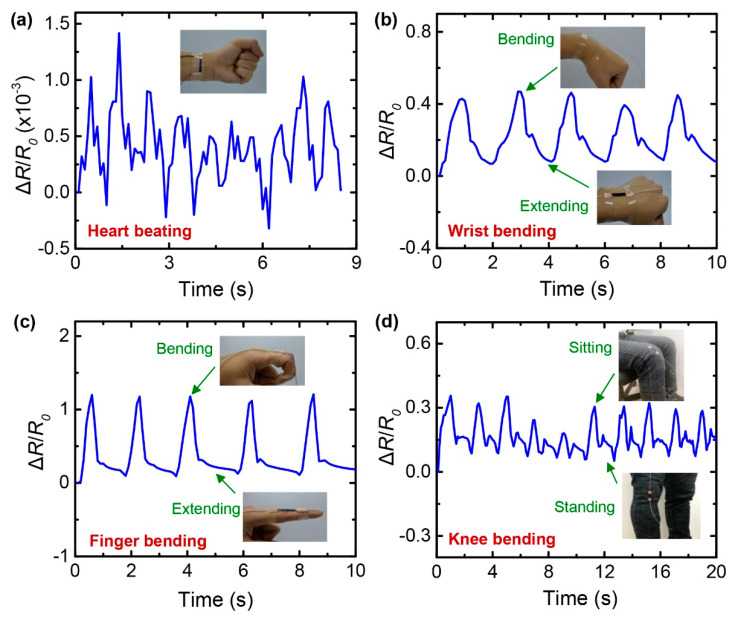
Real-time monitoring of various human motions using stretchable G/AgNW/SpX composite strain sensors. Resistance responses of the sensors clearly indicate: (**a**) heartbeats at the wrist; (**b**) bending of the wrist; (**c**) bending of the finger; (**d**) movement of the knee joint.

The high-performance stretchable strain sensors employing the G/AgNW/SpX composite can be utilized for various applications. Figure 9 presents the application of the strain sensor for the detection of human motions. Figure 9a demonstrates the detection of heartbeats by firmly placing the sensor on the skin of the wrist. A periodic waveform with 10 pulses in 8.5 s was sensed, indicating a heartbeat rate of approximately 71 beats per minute. This proves that the G/AgNW/SpX strain sensor is sufficiently sensitive to detect considerably subtle human motions. Furthermore, the sensor was placed on the skin of the wrist to detect the movement of the wrist (Figure 9b). When the wrist was bent, there was an abrupt change in the relative resistance due to the stretching of the sensor. When the wrist was extended, the resistance responses assumed their original state. Similarly, the strain sensor could evidently detect finger movements when it was placed on the index finger (Figure 9c). Additionally, the sensor could also be incorporated in clothes to detect large human body motions. Figure 9d shows the case wherein the sensor was attached to the cloth at the knee for the purpose of monitoring seating and standing motions. Based on these demonstrations, the strain sensor employing the G/AgNW/SpX composite exhibits a significant potential for the detection of human motion, over a wide range of strain.

## 4. Conclusions

To develop cheap and reliable strain sensors featuring high stretchability, high sensitivity, low hysteresis, and high durability, stretchable conductive polymer composites containing hybrid fillers of graphene and AgNWs were fabricated by incorporating the fillers into spandex. The synergistic effect of 2D graphene and 1D AgNWs in the composite improved the electrical conductivity and mechanical stretchability with low hysteresis, as compared to the composite containing graphene alone. Stretchable strain sensors fabricated using this composite were capable of detecting a strain of up to 120%, with a GF of 150.3. Additionally, these sensors had high cyclability over 1000 cycles and the ability to detect slow and fast deformation, under a strain rate ranging from 1 to 20%/s. Furthermore, these high-performance stretchable strain sensors were used for monitoring human body motions by attaching them to the skin or to clothes. They successfully sensed diverse human motions such as heartbeats and the bending of a wrist, finger, and knee joint due to their high sensitivity and large strain range. This indicates the significant potential of G/AgNW/SpX stretchable strain sensors for a wide variety of future applications including healthcare engineering, sport performance monitoring, soft robotics, and virtual reality.
